# HIV-1 Drug Resistance among Treatment-Naïve Patients in Russia: Analysis of the National Database, 2006–2022

**DOI:** 10.3390/v15040991

**Published:** 2023-04-18

**Authors:** Alina Kirichenko, Dmitry Kireev, Ilya Lapovok, Anastasia Shlykova, Alexey Lopatukhin, Anastasia Pokrovskaya, Marina Bobkova, Anastasiia Antonova, Anna Kuznetsova, Ekaterina Ozhmegova, Sergey Shtrek, Aleksej Sannikov, Natalia Zaytseva, Olga Peksheva, Michael Piterskiy, Aleksandr Semenov, Galina Turbina, Natalia Filoniuk, Andrey Shemshura, Valeriy Kulagin, Dmitry Kolpakov, Aleksandr Suladze, Valeriya Kotova, Lyudmila Balakhontseva, Vadim Pokrovsky, Vasiliy Akimkin

**Affiliations:** 1 Central Research Institute of Epidemiology, 111123 Moscow, Russia; dmitkireev@yandex.ru (D.K.); i_lapovok@mail.ru (I.L.); murzakova_a.v@mail.ru (A.S.); a.lopatukhin@gmail.com (A.L.); pokrovskaya_av@mail.ru (A.P.); pokrovsky.vad@yandex.ru (V.P.); vgakimkin@yandex.ru (V.A.); 2 Department of Infectious Diseases with Courses of Epidemiology and Phthisiology, Medical Institute, Peoples’ Friendship University of Russia (RUDN University), 117198 Moscow, Russia; 3 Gamaleya National Research Center for Epidemiology and Microbiology, 123098 Moscow, Russia; mrbobkova@mail.ru (M.B.); anastaseika95@mail.ru (A.A.); a-myznikova@list.ru (A.K.); belokopytova.01@mail.ru (E.O.); 4 Omsk Research Institute of Natural Focal Infections, 644080 Omsk, Russia; studi1990@mail.ru (S.S.); sannikov.1.96@mail.ru (A.S.); 5 Department of Microbiology, Virology and Immunology, Omsk State Medical University, 644099 Omsk, Russia; 6 Academician I.N. Blokhina Nizhny Novgorod Scientific Research Institute of Epidemiology and Microbiology of the Rospotrebnadzor, 603022 Nizhny Novgorod, Russia; vtashca@mail.ru (N.Z.); peolinn@mail.ru (O.P.); 7 Federal Scientific Research Institute of Viral Infections «Virome» Federal Service for Surveillance on Consumer Rights Protection and Human Wellbeing, 620030 Ekaterinburg, Russia; piterskiy_mv@eniivi.ru (M.P.); semenov_av@eniivi.ru (A.S.); 8 Lipetsk Regional Center for Prevention and Control of AIDS and Infectious Diseases, 398043 Lipetsk, Russia; galinaturbina@yandex.ru (G.T.); natafilonyuk@mail.ru (N.F.); 9 Clinical Center of HIV/AIDS Treatment and Prevention of the Ministry of Health of Krasnodar Region, 350000 Krasnodar, Russia; shemsh@mail.ru (A.S.);; 10 Department of Infectious Diseases and Epidemiology, The Faculty of Advanced Training and Professional Retraining of Specialists, Kuban State Medical University of the Ministry of Health of the Russian Federation, 350063 Krasnodar, Russia; 11 Rostov Research Institute of Microbiology and Parasitology, 344000 Rostov-on-Don, Russia; dimakolpakov@mail.ru (D.K.); sualrostov@mail.ru (A.S.); 12 Khabarovsk Research Institute of Epidemiology and Microbiology of the Rospotrebnadzor, 680610 Khabarovsk, Russia; kotova.valeriya@mail.ru (V.K.);

**Keywords:** HIV-1, drug resistance, treatment-naïve, Russia, database

## Abstract

In Russia, antiretroviral therapy (ART) coverage has significantly increased, which, in the absence of routine genotyping testing, could lead to an increase in HIV drug resistance (DR). The aim of this study was to investigate the patterns and temporal trends in HIV DR as well as the prevalence of genetic variants in treatment-naïve patients from 2006 to 2022, using data from the Russian database (4481 protease and reverse transcriptase and 844 integrase gene sequences). HIV genetic variants, and DR and DR mutations (DRMs) were determined using the Stanford Database. The analysis showed high viral diversity, with the predominance of A6 (78.4%), which was the most common in all transmission risk groups. The overall prevalence of surveillance DRMs (SDRMs) was 5.4%, and it reached 10.0% in 2022. Most patients harbored NNRTI SDRMs (3.3%). The prevalence of SDRMs was highest in the Ural (7.9%). Male gender and the CRF63_02A6 variant were association factors with SDRMs. The overall prevalence of DR was 12.7% and increased over time, primarily due to NNRTIs. Because baseline HIV genotyping is unavailable in Russia, it is necessary to conduct surveillance of HIV DR due to the increased ART coverage and DR prevalence. Centralized collection and unified analysis of all received genotypes in the national database can help in understanding the patterns and trends in DR to improve treatment protocols and increase the effectiveness of ART. Moreover, using the national database can help identify regions or transmission risk groups with a high prevalence of HIV DR for epidemiological measures to prevent the spread of HIV DR in the country.

## 1. Introduction

Despite continued efforts, the HIV/AIDS epidemic remains a major public health concern worldwide, including in Russia. At the end of 2021, the ECDC and WHO reported that Russia has the highest rates of newly diagnosed HIV infections in the European region (40.2 per 100,000 population) [1]. According to the national estimation, this rate is higher and in 2021 amounted to 48.7 per 100,000 population [2]. In 2021, the number of diagnosed people living with HIV (PLWH) was 1,137,596, which was 0.8% of the total population of the country and 1.5% of those aged 15–49 [2].

Previously, the HIV epidemic in Russia was driven primarily by the spread of the virus among intravenous drug users (IDUs), making up 56.9% of all HIV cases. However, the epidemic has changed in the last 5 years. Heterosexual transmission has become the main one, being the transmission route of 67.8% of new HIV cases in 2021. There has been an increase in the prevalence of HIV infection among men having sex with men (MSM) (3.0% in 2021) [2].

One of the most effective measures to reduce the epidemic is antiretroviral therapy (ART), which has shown significant improvements in morbidity and mortality rates over the years. Due to the use of ART, it is possible to increase the life expectancy and improve the quality of life of HIV-infected individuals, as well as preventing onward transmission [3,4,5,6].

In Russia, the scaling up of ART started in 2006 during the national health program. To date, 40 international non-proprietary names of drugs for HIV infection treatment have been registered in Russia. Until 2020, according to the clinical recommendations of the Ministry of Health, the main first-line ART regimens were based on two nucleoside reverse transcriptase inhibitors (NRTIs)—mainly tenofovir disoproxil fumarate (TDF) or zidovudine (AZT) (until 2014) + lamivudine (3TC) or emtricitabine (FTC) + one non-nucleoside reverse transcriptase inhibitor (NNRTI)–efavirenz (EFV) [7]. To date, a national guideline has transitioned to two NRTIs- and NNRTI- or dolutegravir (DTG)-containing first-line ART regimens. The current preferred first-line ART regimens contain TDF + 3TC or FTC + EFV or DTG or elsulfavirine (ESV) [8]. ESV is an NNRTI, which was developed and registered in Russia in 2017 [9]. 

In practice, in 2021, 24% of patients received DTG as a third component, the same number received EFV, and 17% received lopinavir/ritonavir (LPV/r). TDF was the dominant drug among NRTIs, accounting for 78% [10].

The proportion of PLWH who received ART from the total diagnosed PLWH increased from 4% in 2006 to 58% in 2021, and the total number of PLWH receiving ART has exceeded 600,000 [10,11].

In 2021, the percentage of PLWH who achieved an undetectable HIV viral load (VL) was 46.4%, and it was 79.9% among those receiving ART [10]. It has been shown that achieving the third target of “95-95-95” for viral suppression is key to reducing the rate of new HIV infections [12]. One of the main reasons (70–80%) for ART virological failure is HIV drug resistance (DR) [13].

While in high-income countries HIV DR testing is performed for all patients with virologic failure and all patients initiating ART, in Russia, genotypic testing is rare due to its weak laboratory capacity and high cost. According to the national standard of primary health care, DR testing is recommended for 10% of patients during treatment twice annually, and there are no recommendations for treatment-naïve patients [14].

However, the genotyping test is performed for less than 10% of patients with virologic failure [15,16], and in rare cases, for treatment-naïve patients.

The studies of HIV DR in treatment-naïve patients in Russia were conducted unevenly in space and time, the sample sizes were insufficient, and the authors used different approaches (surveillance drug resistance mutations (SDRMs) list [17], clinical interpretation, different accounting for polymorphism mutations).

Across the most representative HIV DR studies in treatment-naïve patients, the following ones are notable. A study conducted in Moscow and the Moscow region (527 patients) in 2008–2017 estimated that the SDRM prevalence was 2.0% [18]. A study in the south of Russia (323 patients) in 2014–2019 reported a higher SDRM prevalence of 4.6% [19]. Similar data were found in nationwide studies where the prevalence of SDRMs was 5.3% (1560 patients, 1998–2017 sampling years) [20] and 4.5% of pretreatment DR (465 patients, 2017–2019 sampling years) [21]. Data on the prevalence of DR or DRMs to integrase strand transfer inhibitors (INSTIs) are very sparse. The study with the largest number of participants (225 patients, 2007–2019 sampling years) showed that DR to INSTI was 1.3% [22].

Most other studies have been conducted with samples of fewer than 50 patients. In this regard, HIV DR data in Russia are fragmented and incomparable.

With the unavailability of baseline DR testing for all patients, surveillance databases could resolve the data fragmentation problem, such as the Russian national database (RuHIV) (https://ruhiv.ru/, accessed on 1 December 2022), which was created in 2009 to monitor the emergence and transmission of HIV DR and circulating HIV-1 genetic variants in the country. The new Sanitary Rules and Regulations [23] stipulate that the genotyping laboratory should submit the results of HIV DR testing since 2021 to this database. The database is currently the largest national database of HIV genotypes in the country. It contains 13,126 sequences from 10,626 HIV-infected Russian individuals living in all federal districts (FDs) (on 1 December 2022). Sequences are linked with clinical (stage of HIV infection, ART history, treatment adherence, etc.), epidemiological (HIV transmission routes, region of origin, etc.), demographic (age, sex, etc.), and laboratory data (date of first positive immune blot, VL, CD4+ T-cell count, etc.).

In this study, our aim was to investigate the patterns and temporal trends in HIV DR as well as the prevalence of HIV genetic variants in treatment-naïve patients since the scaling up of ART in Russia from 2006 to 2022, using the largest available dataset from the Russian national database (https://ruhiv.ru/, accessed on 1 December 2022).

## 2. Materials and Methods

### 2.1. Study Population and Data Collection

We analyzed sequences and linked demographic, clinical, and epidemiological data uploaded to the RuHIV database (https://ruhiv.ru/, accessed on 1 December 2022) from 4481 treatment-naïve HIV-infected patients. The sequences covered the HIV-1 protease (PR), and part of the reverse transcriptase (RT) was available for all patients; additionally, sequences covering the HIV-1 integrase (INT) were available for 844 of them.

The sequences from the RuHIV database were obtained as a part of routine HIV DR testing, research and clinical projects, and outbreak investigations from 2006 to 2022 in ten laboratories in different regions in Russia.

In order to avoid distortion of the results, in the case of multiple available sequences for one patient, the nucleotide sequences from the earliest blood sampling were used.

### 2.2. RNA Extraction and HIV-1 Sequencing

Commercial genotyping kits (the AmpliSens^®®^ HIV-Resist-Seq kit (Central Research Institute of Epidemiology, Russia), the ViroSeq™ HIV-1 Genotyping System kit (Celera Diagnostics, Alameda, CA, USA)), and in-house methods were used for RNA extraction from the blood plasma samples and Sanger-based or NGS-based (with 20% detection threshold) sequencing of the HIV pol gene regions encoding the PR-RT (2253–3369 bp according to the HXB-2 strain, GenBank accession number K03455), and the INT (4230–5093 bp according to HXB-2, GenBank accession number K03455).

### 2.3. Sequence Quality Control

Quality assurance of HIV-1 sequences was carried out using the WHO BCCfE HIVDR QC tool (http://pssm.cfenet.ubc.ca/who_qc/, accessed on 1 December 2022) and quality control tool of Calibrated Population Resistance (CPR) (https://hivdb.stanford.edu/cpr/, accessed on 1 December 2022) before data analysis. The sequences, identified as failing in at least one tool, were excluded from the analysis. All sequence pairs with a genetic distance <0.5% within the same sequencing batch were excluded.

### 2.4. HIV-1 Subtyping

HIV-1 genetic variants were determined using PR-RT sequences by the Stanford HIV Drug Resistance Database (https://hivdb.stanford.edu/, accessed on 10 December 2022) and subsequently clarified by the HIV BLAST tool (https://www.hiv.lanl.gov/content/sequence/BASIC_BLAST/basic_blast.html, accessed on 10 December 2022).

### 2.5. HIV-1 Drug Resistance Interpretation

The Stanford HIV Drug Resistance Database (HIVdb Program v 9.4 and Calibrated Population Resistance Tool) was used to describe and interpret the HIV DR level and DRMs, including SDRMs [17].

The DR level was classified according to the Stanford Penalty Score as high (60), intermediate (30–59), or low (15–29) to:

- nucleoside reverse transcriptase inhibitors (NRTIs): abacavir (ABC), zidovudine (AZT), emtricitabine (FTC), lamivudine (3TC), tenofovir (TDF), stavudine (d4T), didanosine (ddI);
- non-nucleoside reverse transcriptase inhibitors (NNRTIs): doravirine (DOR), efavirenz (EFV), etravirine (ETR), nevirapine (NVP), rilpivirine (RPV);
- protease inhibitors (PIs): atazanavir (ATV), darunavir (DRV), lopinavir (LPV), fosamprenavir (FPV), indinavir (IDV), nelfinavir (NFV), saquinavir (SQV), tipranavir (TPV);
- integrase strand transfer inhibitors (INSTIs): bictegravir (BIC), cabotegravir (CAB), dolutegravir (DTG), elvitegravir (EVG), raltegravir (RAL).

Sequences with DR were defined as sequences with a Stanford Penalty Score of 15 or higher.

### 2.6. Statistical Analysis

Estimates of the prevalence of DR and DRMs were calculated with 95% confidence intervals (CIs). Fischer’s exact test was used for the analysis of differences between proportions. A two-tailed *p*-value < 0.05 was considered significant. All analyses were performed using STATA (v 15).

### 2.7. Ethics

This study was approved by the Ethics Review Committee of the Central Research Institute of Epidemiology (Moscow, Russia).

The informed written consent of each HIV-infected patient or the patient’s legal guardian was obtained prior to the sampling and collection of clinical, demographic, and epidemiological data. All the data were anonymized and coded at a national level before being uploaded to the RuHIV database.

## 3. Results

### 3.1. Characteristics of the Study Population

We studied the genotype results of 4481 HIV-1-infected treatment-naïve patients from all Russian FDs. Most of the study patients were from the Central FD (1661; 37.1%), followed by Southern (666; 14.9%), and Volga (617; 13.8%) FDs (Table 1). The smallest number of patients with available genotypes was from the North Caucasian FD (18; 0.4%).

Of the 4481 study patients, the majority were male (2510; 56.0%) and reported heterosexual contact as the main risk factor (1951; 43.5%), followed by IDU (967; 21.6%). 

The median age of the patients was 33 years (range 27–39). The study population included 20 (0.4%) HIV-infected individuals aged <18 years.

All patients had detectable VL at genotyping test. The median plasma HIV-1 RNA was 4.6 log10 copies/mL (range 4.0–5.2), and the median CD4+ T-cell count was 401 cells/mm^3^ (range 264–559.5).

The years of diagnosis based on first HIV-positive immune blotting ranged between 1997 and 2022, and blood sampling years ranged between 2006 and 2022.

### 3.2. Prevalence of HIV-1 Genetic Variants

The vast majority of the study PR-RT sequences (n = 4481) were classified as sub-subtype A6 (3514; 78.4%). The circulating recombinant form (CRF) 63_02A6 (414; 9.2%), B (295; 6.6%), CRF03_A6B (73; 1.6%), CRF02_AG (70; 1.6%), and G (58; 1.3%) were also detected at low prevalence. In addition, 57 (1.3%) sequences were found to belong to other genetic variants, including CRF01_AE, A1, 19_cpx, 14_BG, CRF01_AE/B, CRF20_BG, 56_cpx, C, 11_cpx, CRF06_cpx, and F1, each with a prevalence of less than 1%.

**Figure 1:**
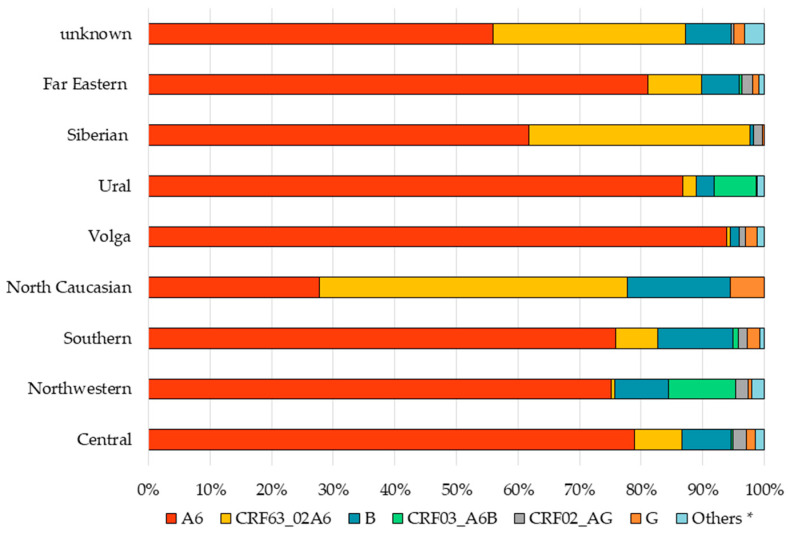
Prevalence of HIV-1 genetic variants in the FDs of Russia. * Others (other HIV-1 genetic variants) include CRF01_AE, A1, 19_cpx, 14_BG, CRF01_AE/B, CRF20_BG, 56_cpx, C, 11_cpx, CRF06_cpx, and F1. Abbreviation: CRF, circulating recombinant form.

We analyzed the prevalence of HIV-1 genetic variants in patients living in different FDs (Figure 1), across time (Figure S1) and belonging to different transmission risk groups (Table 2).

Sub-subtype A6 was the most common genetic variant circulating among residents of all FDs. The exception was the study patients from the North Caucasian FD, who were mainly infected with CRF63_02A6, which does not reflect the actual situation due to the small sample size (n = 18). In the Siberian FD, there was also a high prevalence of CRF63_02A6 (36.0%). In the Ural and Northwestern FDs, the prevalence of CRF03_A6B was 6.7% and 10.8%, respectively, which is higher than in other districts (0–1.6%) (*p* < 0.0001). The highest genetic diversity was observed in the Central, Northwestern, and Southern FDs.

Due to the small sample size in some years of blood sampling, we combined data to analyze temporal trends of HIV genetic variants. The temporal trends of sub-subtype A6 have decreased, whereas CRF63_02A6, CRF02_AG and other genetic variants (CRF01_AE, A1, 19_cpx, 14_BG, CRF01_AE/B, CRF20_BG, 56_cpx, C, 11_cpx, CRF06_cpx, F1) have been on an increasing trend since 2012 (Figure S1). The proportion of subtype B has not changed since 2009. The proportion of CRF03_A6B and subtype G did not change throughout the study period.

Sub-subtype A6 was the most common genetic variant among patients belonging to all transmission risk groups. In addition to sub-subtype A6, subtype B was also noted among men with sexual transmission without clarification (A6: 71.8%; B: 15.3%). Among men having sex with men (MSM), a high frequency of subtype B was also found (A6: 51.0%; B: 33.7%), and among IDUs: CRF63_02A6 (A6: 81.6%; CRF63_02A6: 12.5%).

### 3.3. Prevalence of Major DRMs and SDRMs

We studied the prevalence of all major DRMs and SDRMs using PR-RT (n = 4481) and INT (n = 844) sequences for NRTIs, NNRTIs, PIs, and INSTIs, respectively.

The overall prevalence of SDRMs in treatment-naïve patients with the 2006–2022 sampling years was 5.4% (95% CI, 4.7–6.1%). Most of these patients harbored SDRMs to NNRTIs (3.3% (95% CI, 2.8–3.8%)), followed by NRTIs (1.4% (95% CI, 1.0–1.7%)), PIs (1.4% (95% CI, 1.0–1.7%)), and INSTIs (0.1% (95% CI, 0.0–0.7%)).

The most frequent NNRTI SDRMs were K103N/S (100/4481; 2.2%) and G190A/S (27/4481; 0.6%), which cause high-level resistance to both EFV and NVP, and K101E (17/4481; 0.4%), which confers resistance to all NNRTIs. For NRTIs, M184V/I (30/4481; 0.7%) and T215D/Y/S/I/E (15/4481; 0.3%) were the most commonly observed SDRMs. M184V/I was associated with DR to 3TC, FTC, and ABC. T215Y confers a high level of DR to AZT, and other revertant mutations in this position do not reduce NRTI susceptibility but arise from viruses that contain T215Y/F. Of the PI SDRMs, M46I/L (28/4481; 0.6%) and I85V (14/4481; 0.3%) were the most frequently found, which individually have minimal effects on PI susceptibility. For INSTIs, only one SDRM was found, R263K (1/844; 0.1%), which reduced the susceptibility of the virus to all drugs of this class [24].

The most common DRM was A62V (1787/4481; 39.9%), which is non-SDRM and well-known as polymorphic in sub-subtype A6 and, alone, probably confers little to no NRTI resistance. The most prevalent NNRTI-associated DRM included E138A (239/4481; 5.3%), which is also polymorphic in sub-subtype A6 and associated with reduced susceptibility to RPV. Other commonly observed NNRTI DRMs included V179D/E/T (70/4481; 1.6%) and V106I (57/4481; 1.3%), which are associated with minimal reduction in NNRTI susceptibility [24].

The prevalence of all DRMs, including SDRMs, is shown in Table S1.

**Figure 2:**
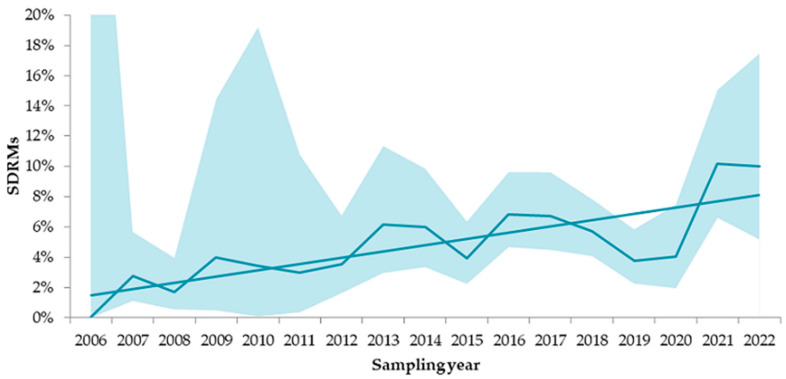
Temporal trends in SDRMs in Russia in the 2006–2022 sampling years. The range of the 95% confidence interval is represented as a blue cloud around a line. Abbreviation: SDRMs, surveillance drug resistance mutations.

The yearly prevalence of SDRMs varied from 2006 to 2022 but generally increased over time (Figure 2). The highest prevalence of SDRMs was determined in patients in the 2021 and 2022 sampling years: 10.2% (95% CI, 6.6–15.1%) and 10.0% (95% CI, 5.2–17.5%), respectively.

Additionally, we explored the association between SDRMs and the demographic, epidemiological, and clinical characteristics of patients. Statistical analysis revealed that SDRMs were observed significantly more often in male patients than female patients (6.1% vs. 4.5%, *p* = 0.0181) and patients infected with CRF63_02A6 compared to other variants (11.4% vs. 5.0%, *p* < 0.0001), whereas patients infected with sub-subtype A6 are less likely to have SDRMs than those infected with other variants (4.7% vs. 8.8%, *p* < 0.0001). There were no significant associations between the SDRMs in study patients and the median age, transmission risk group, VL, and CD4+ T-cell count. 

**Figure 3:**
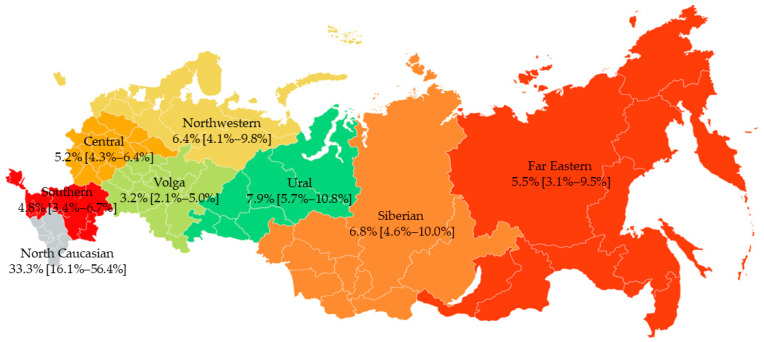
Prevalence of SDRMs in FDs of Russia in the 2006–2022 sampling years. The range of the 95% confidence interval is enclosed in square brackets.

However, significant differences were found in the prevalence of DR across FDs (Figure 3). The prevalence of SDRMs was significantly higher in the North Caucasian (33.3% (95% CI, 6.1–56.4%)) (*p* = 0.0003) and Ural (7.9% (95% CI, 5.7–10.8%)) (*p* = 0.0376) FDs. Conversely, the prevalence of SDRMs was significantly lower in the Volga FD (3.2% (95% CI, 2.1–5.0%)) (*p* = 0.0247).

It is important to clarify that the extremely high prevalence of SDRMs in the North Caucasian FD was related to both a small sample (n = 18) and bias. For six patients from this FD, sequences were obtained during the investigation of a nosocomial outbreak; all of them had identical viruses with K103S SDRM.

### 3.4. Prevalence of DR

We studied the prevalence of DR to all ART drugs using PR-RT (n = 4481) and INT (n = 844) sequences for NRTIs, NNRTIs, PIs, and INSTIs, respectively.

**Figure 4:**
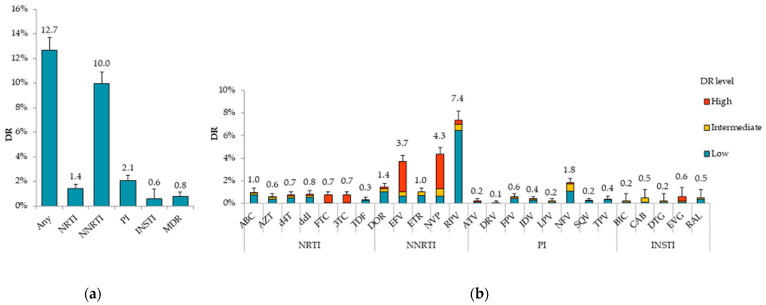
HIV DR prevalence in Russia in the 2006–2022 sampling years by (**a**) drug class and (**b**) antiretroviral drugs with DR levels. PR-RT (n = 4481) and INT (n = 844) sequences were used to analyze DR to NRTIs, NNRTIs, PIs, and INSTIs, respectively. Error bars represent 95% CIs. Abbreviations: NRTI, nucleoside reverse transcriptase inhibitor; NNRTI, non-nucleoside reverse transcriptase inhibitor; PI, protease inhibitor; INSTI, integrase strand transfer inhibitor; MDR, multidrug-resistance; ABC, abacavir; AZT, zidovudine; FTC, emtricitabine; 3TC, lamivudine; TDF, tenofovir; d4T, stavudine; ddI, didanosine; DOR, doravirine; EFV, efavirenz; ETR, etravirine; NVP, nevirapine; RPV, rilpivirine; ATV, atazanavir; DRV, darunavir; LPV, lopinavir; FPV, fosamprenavir; IDV, indinavir; NFV, nelfinavir; SQV, saquinavir; TPV, tipranavir; BIC, bictegravir; CAB, cabotegravir; DTG, dolutegravir; EVG, elvitegravir; RAL, raltegravir.

The overall prevalence of DR to any drug classes in the 2006–2022 sampling years was 12.7% (95% CI, 11.6–13.7%). Considering individual drug classes, the prevalence of DR was 10.0% (95% CI, 9.1–10.9%) to any NNRTIs, 1.4% (95% CI, 1.1–1.8%) to any NRTIs, 2.1% (95% CI, 1.7–2.5%) to any PIs, and 0.6% (95% CI, 0.2–1.4%) to any INSTIs (Figure 4a).

Less than 1% of study patients were found with multidrug resistance (MDR) (0.8% (95% CI, 0.6–1.1%)): NRTI + NNRTI (0.5%), PI + NNRTI (0.2%), PI + NRTI (0.1%), and PI + NNRTI + NNRTI (0.02%).

NNRTI DR to single drugs was highest to RPV (7.4%) with low DR level. The DR to NVP and EFV was 4.3% and 3.7%, respectively, and the DR level was mostly categorized as high. Notably, the DR to NVP/EFV in all FDs, except for the North Caucasian FD due to sampling bias, did not exceed 10% and was the highest in the Ural FD (5.6% to NVP, 4.9% to EFV) (Table S2).

Less than 1% of the viruses were resistant to any of the PIs with the exception of NFV, to which DR was found in 1.8% of treatment-naïve patients. The prevalence of DR to NRTIs and INSTIs did not exceed 1% for each drug individually (Figure 4b).

**Figure 5:**
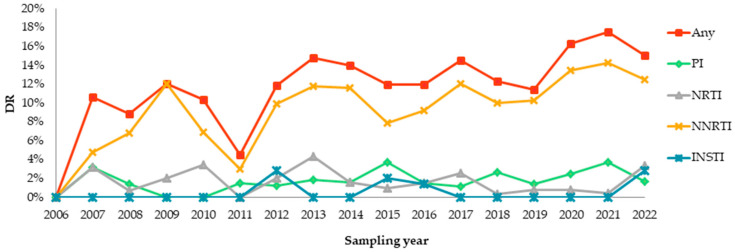
Temporal trends in HIV DR in Russia in the 2006–2022 sampling years by drug class. PR-RT (n = 4481) and INT (n = 844) sequences were used to analyze DR to NRTIs, NNRTIs, PIs, and INSTIs, respectively. Abbreviations: PI, protease inhibitor; NRTI, nucleoside reverse transcriptase inhibitor; NNRTI, non-nucleoside reverse transcriptase inhibitor; INSTI, integrase strand transfer inhibitor.

DR presented an overall increasing trend between the 2006 and 2022 sampling years, which was driven primarily by NNRTI resistance (Figure 5). The DR to PIs, NRTIs, and INSTIs showed no trends and was low across all years, never exceeding 4%. 

## 4. Discussion

The HIV epidemic in Russia continues to pose a threat to public health. Wide use of ART has substantially reduced HIV-related morbidity, mortality, and HIV transmission [3,4,5,6]. The number of PLWHs receiving ART in Russia has been constantly growing since the ART national program started in 2006, and in 2021, it reached 58% [10]. However, the ART coverage is far from achieving the levels needed to control the HIV epidemic and remains unsatisfactory due to the large number of newly diagnosed cases [25]. Therefore, it is expected that ART coverage among those who are HIV-positive will significantly increase.

Unfortunately, the expanding ART coverage, especially in the absence of routine HIV DR testing, can lead to an increase in the HIV DR prevalence [26,27]. The presence of HIV DR is associated with poorer virological outcomes on first-line ART [28,29], which could contribute to the further emergence of DR and increasing mortality, HIV morbidity, and the cost of epidemic control programs [26,30,31,32]. Therefore, knowledge of HIV DR patterns and trends is critical to effectively treating patients at both individual and national levels.

Furthermore, in Russia, there is a problem of the coverage of PLWH with HIV VL and DR tests that can lead to an increase in HIV DR and jeopardize ART success. In addition to limited access to laboratory monitoring of the treatment efficacy, there are some other prerequisites for the emergence of HIV DR, such as high HIV prevalence, poor adherence to ART, and the use of NNRTI-based regimens with a low genetic barrier.

Whereas baseline HIV genotyping is not available in Russia, as well as HIV DR surveillance studies at the country level, centralized collection and analysis of all received HIV nucleotide sequences can help understand the patterns and trends in DR.

In the current study, we investigated the patterns and temporal trends in HIV-1 DR as well as the prevalence of genetic variants in treatment-naïve patients since the scaling up of ART in Russia started in 2006 using data from the Russian national database (https://ruhiv.ru/, accessed on 1 December 2022). To the best of our knowledge, this is the largest HIV DR study in Russia, including 4481 treatment-naïve patients (4481 PR-RT and 844 INT sequences), covering the longest period (17 years). The number of analyzed sequences exceeds the number of available genotypes from Russian treatment-naïve patients in The Los Alamos HIV Sequence Database (https://www.hiv.lanl.gov/content/index, accessed on 1 December 2022) and is linked with full demographic, clinical, and epidemiological data.

The present study showed that the majority of patients within the 2006–2022 sampling years reported heterosexual contact as the main risk factor (43.5%), followed by IDU (21.6%) and MSM (6.8%). This reflects the main trends in the epidemic, including an increase in the proportion in the transmission risk group of heterosexuals and MSM, and a decrease in IDUs [2].

While the proportion of transmission risk groups has changed over time, so has the distribution of genetic variants within them. Our data revealed that sub-subtype A6 (formerly FSU-A or IDU-A) (78.4%), whose distribution began in the mid-1990s in Odessa, Ukraine, with an outbreak among IDUs [33,34,35], remains the dominant HIV-1 genetic variant in all parts of Russia. Although nowadays this genetic variant is actively spreading not only among IDUs (81.6%) but also among all transmission risk groups (sexual without clarification (71.8%), heterosexual (84.5%), MSM (51.0%), mother-to-child (94.4%), and outbreak (69.6%)), this could be a consequence of intermingling between them. The recent study [36] has already demonstrated the high distribution of sub-subtype A6 among IDUs as well as heterosexuals and MSM.

The following identified genetic variant was CRF63_02A6 (9.2%), which has spread in the Central Asian countries [37] and the Siberian FD [38]. According to our data, this genetic variant was also most common among patients living in the Siberian FD (36.0%) and among IDUs (12.5%).

One of the commonly observed genetic variants was subtype B (6.6%), with the highest prevalence among MSM (33.7%). Other genetic variants were found with a frequency of less than 5%.

Previous studies report that sub-subtype A6 is also the predominant or main genetic variant in the former Soviet Union countries [21,36], reflecting economic and cultural relationships between countries, whereas subtype B dominates in European countries, and subtype A accounts for less than 10% [39].

Overall, the analysis of HIV-1 genetic variants in Russia showed a high genetic diversity of the virus with multiple subtypes and CRFs, especially in the Central, Northwestern, and Southern FDs. In isolated cases (1.3%), genetic variants atypical for Russia were found, including A1, 19_cpx, CRF14_BG, CRF20_BG, 56_cpx, C, 11_cpx, CRF06_cpx, and F1.

In addition, the increase in the proportion of CRF63_02A6 and CRF02_AG, and the decrease in the proportion of the A6 subtype in the Russian epidemic, previously described for Russia and the countries of the former Soviet Union countries [40,41], is shown.

Our study demonstrated a moderate level of SDRM prevalence (5.4%) in treatment-naïve Russian patients throughout the 2006–2022 study period, which was similar (5.3%) to that observed in the previous nationwide study during the 1998–2017 sampling years [20]. However, we show a constantly increasing rate of SDRMs. The prevalence of SDRMs reached 10.2% and 10.0% in the 2021 and 2022 sampling years, respectively, suggesting the need for continuous surveillance.

The highest SDRM prevalence was observed for NNRTIs (3.3%). This fact may reflect the widespread use of NNRTI-containing regimens and their low genetic barrier. Additionally, we found a low prevalence of SDRMs to NRTIs (1.4%), PIs (1.4%), and INSTIs (0.1%).

Of the SDRMs, K103N/S (2.2%) was the most frequently observed. That fact can be related to the wide use of EFV with low genetic barriers as a part of first-line ART regimens or the result of effective transmission due to the high ability of variants with these mutations to persist for years [42]. Other SDRMs were found in less than 1% of the study patients.

A significant proportion of patients harbored the A62V NRTI mutation (39.9%), which is polymorphic in sub-subtype A6, and it does not cause DR but contributes to its development. Another polymorphic mutation in sub-subtype A6 E138A (5.3%) was the most common NNRTI mutation detected in treatment-naïve Russian patients. In vitro data from site-directed mutagenesis showed that E138A causes a two-fold reduction in susceptibility to RPV [43,44]; however, this does not appear to affect the effectiveness of RPV-containing treatment in real practice [45,46].

The prevalence of SDRMs was uneven across Russia, with the highest in the Ural FD (7.9%) and lowest in the Volga FD (3.2%). It is critical to state that the high prevalence of SDRM found in the North Caucasian FD is a result of the small sample size (n = 18) and the bias of this sample, 33.3% of which consisted of patients infected with the same DR virus variants during a nosocomial outbreak. Thus, future DR surveillance studies in the Ural FD due to the highest rate of SDRMs, as well as in the North Caucasian FD due to the small sample and bias, are necessary.

Additionally, we have shown factors that are associated with an increased risk of SDRMs: male gender and the CRF63_02A6 genetic variant. 

In the present study, the overall prevalence of DR to any drug classes in the 2006–2022 sampling years was 12.7%, mainly due to NNRTI DR (10.0%). Furthermore, we noted a temporal trend in the spread of DR due to NNRTI DR. The predominance of NNRTI DR is related not only with widespread and longer use of these drugs in Russia and the low genetic barrier of drugs, but also with features of the A6 genetic variant. Thus, most of the patients with NNRTI DR have DR to RPV (7.4%), mainly due to polymorphic mutation E138A.

Most importantly, the DR to NVP and EFV was 4.3% and 3.7%, respectively, and in all FDs, except for the North Caucasian FD due to sampling bias, it did not reach 10%, the threshold defined by the WHO at which to recommend urgent public health action, including the implementation of an NNRTI-free first-line regimen [47,48].

We show that the prevalence of DR to PIs and NRTIs was low, respectively, 2.1% and 1.4%. The prevalence of DR to INSTIs was even lower, at 0.6% overall. Thus, we suggest that routine DR testing to INSTIs prior to treatment is currently not required in Russia.

Additionally, our analysis revealed that the prevalence of DR to first-line ART-preferred regimen drugs in Russia was low: TDF (0.3%) + 3TC (0.7%) or FTC (0.7%) + EFV (3.7%) or DTG (0.2%). Thus, the current ART regimen among treatment-naïve patients can be expected to be effective.

Notably, most of the patients harbored an HIV variant with DR to only one drug class; triple-class DR was observed in 0.02% of treatment-naïve patients.

Previous studies in treatment-naïve patients from the former Soviet Union countries, where baseline genotyping is also not available, showed a prevalence of pretreatment DR between 2.8% in Uzbekistan and 16.7% in Tajikistan [21]. Among European countries where a long history of ART and high ART coverage, but at the same time, baseline genotyping is a part of routine clinical practice, the overall prevalence of transmitted DR in treatment-naïve patients ranged from 12.8% to 14.5% and presented a decreasing trend [39,49].

One of the limitations presented by the study was the risk of biased assessments due to the dataset. The restricted number of patients from the North Caucasian FD, most of whom consisted of outbreak investigation sequences, skewed the results for the prevalence of genetic variants and HIV DR. We consider that it is also important to analyze sequences obtained not only from routine HIV DR testing but also from clinical trials or outbreak investigations; as long as these patients remain untreated, they are a potential source of new infections. However, increasing the sample size would help offset these problems.

It is also necessary to note the uneven distribution of the sample size across years of blood sampling, with the largest coverage in 2018 (15.2%), and the smallest in 2006 (0.2%).

Despite these sampling limitations, we believe that, until obstacles to the expansion of DR testing in Russia such as the large number of HIV-infected people and insufficient funding are overcome, the analysis of the national database is optimal for understanding the HIV DR patterns and trends. 

The centralized data collection of HIV-1 sequences linked with epidemiological data is crucial for the development of a public health response and can be used not only to understand HIV DR but also for the molecular epidemiology of HIV-1.

Using the national database, it is possible to roughly identify regions or transmission risk groups with a high prevalence of HIV DR and conduct further directed surveillance studies. However, it is necessary to carefully approach the study sample in order to avoid bias in the results.

## Figures and Tables

**Table 1:** Epidemiological and clinical characteristics of the study population.

Characteristic	Total (n = 4481)
Age (years), median (IQR)	33 (27–39)
Sex, n (%)	
Male	2510 (56.0)
Female	1847 (41.2)
Unknown	124 (2.8)
Transmission risk group, n (%)	
Sexual (without clarification)	163 (3.6)
Heterosexual	1951 (43.5)
MSM	306 (6.8)
IDU	967 (21.6)
Mother-to-child	18 (0.4)
Outbreak	23 (0.5)
Unknown	1053 (23.5)
Viral load (log10 copies/mL), median (IQR)	4.6 (4.0–5.2)
CD4+ T-cell count (cells/mm3), median (IQR)	401 (264–559.5)
FD, n (%)	
Central	1661 (37.1)
Northwestern	297 (6.6)
Southern	666 (14.9)
North Caucasian	18 (0.4)
Volga	617 (13.8)
Ural	432 (9.6)
Siberian	353 (7.9)
Far Eastern	217 (4.8)
Unknown	220 (4.9)
Year of diagnosis, n (%)	
1997–1999	61 (1.4)
2000	48 (1.1)
2001	79 (1.8)
2002	43 (1.0)
2003	32 (0.7)
2004	46 (1.0)
2005	50 (1.1)
2006	70 (1.6)
2007	361 (8.1)
2008	201 (4.5)
2009	155 (3.5)
2010	125 (2.8)
2011	144 (3.2)
2012	176 (3.9)
2013	183 (4.1)
2014	340 (7.6)
2015	416 (9.3)
2016	495 (11.0)
2017	384 (8.6)
2018	345 (7.7)
2019	337 (7.5)
2020	107 (2.4)
2021	145 (3.2)
2022	78 (1.7)
Unknown	60 (1.3)
Sampling year, n (%)	
2006	9 (0.2)
2007	254 (5.7)
2008	295 (6.6)
2009	50 (1.1)
2010	29 (0.6)
2011	67 (1.5)
2012	253 (5.6)
2013	162 (3.6)
2014	251 (5.6)
2015	409 (9.1)
2016	469 (10.5)
2017	433 (9.7)
2018	681 (15.2)
2019	508 (11.3)
2020	246 (5.5)
2021	245 (5.5)
2022	120 (2.7)

Abbreviations: IQR, interquartile range; MSM, men having sex with men; IDU, intravenous drug users; FD, federal district.

**Table 2:** Prevalence of HIV-1 genetic variants among the transmission risk groups.

	Genetic Variant, n (%)						
Transmission Risk Group	A6	CRF63_02A6	B	CRF03_A6B	CRF02_AG	G	Others *
Sexual (without clarification)	117 (71.8)	13 (8.0)	25 (15.3)	2 (1.2)	2 (1.2)	4 (2.5)	0
Heterosexual	1648 (84.5)	130 (6.7)	90 (4.6)	31 (1.6)	26 (1.3)	21 (1.1)	5 (0.3)
MSM	156 (51.0)	5 (1.6)	103 (33.7)	1 (0.3)	11 (3.6)	17 (5.6)	13 (4.2)
IDU	789 (81.6)	121 (12.5)	9 (0.9)	21 (2.2)	16 (1.7)	2 (0.2)	9 (0.9)
Mother-to-child	17 (94.4)	1 (5.6)	0	0	0	0	0
Outbreak	16 (69.6)	5 (21.7)	0	0	0	1 (4.3)	1 (4.3)
Unknown	771 (73.1)	139 (13.2)	68 (6.5)	18 (1.7)	15 (1.4)	13 (1.2)	29 (2.8)
All	3514 (78.4)	414 (9.2)	295 (6.6)	73 (1.6)	70 (1.6)	58 (1.3)	57 (1.3)

* Others (other HIV-1 genetic variants) include CRF01_AE, A1, 19_cpx, 14_BG, CRF01_AE/B, CRF20_BG, 56_cpx, C, 11_cpx, CRF06_cpx, F1. Abbreviations: CRF, circulating recombinant form; MSM, men having sex with men; IDU, intravenous drug users.

## Data Availability

The data presented in this study are available on request from the corresponding author. The data are not publicly available due to privacy policy of HIV Russian database.

## Supplementary Materials

The following supporting information can be downloaded at: https://www.mdpi.com/article/10.3390/v15040991/s1, Figure S1: Temporal trends of HIV genetic variants; Table S1: Prevalence of DRMs in Russia in the 2006–2022 sampling years; Table S2: HIV DR prevalence in FDs of Russia in the 2006–2022 sampling years by antiretroviral drugs.

## References

[1] European Centre for Disease Prevention and Control/WHO Regional Office for Europe (2021). HIV/AIDS Surveillance in Europe 2021–2020 Data.

[2] Ladnaia N.N., Pokrovsky V.V., Sokolova E.V., Chekryzhova D.G., Kirzhanova V.V. (2022). Prevalence of human immune deficiency virus infection in the territories of the Russian Federation in 2021. Epidemiol. Infect. Dis. Curr. Items.

[3] Palella F.J., Delaney K.M., Moorman A.C., Loveless M.O., Fuhrer J., Satten G.A., Aschman D.J., Holmberg S.D. (1998). Declining morbidity and mortality among patients with advanced human immunodeficiency virus infection. N. Engl. J. Med..

[4] Cohen M.S., Chen Y.Q., McCauley M., Gamble T., Hosseinipour M.C., Kumarasamy N., Hakim J.G., Kumwenda J., Grinsztejn B., Pilotto J.H. (2011). Prevention of HIV-1 infection with early antiretroviral therapy. N. Engl. J. Med..

[5] Keller S.C., Yehia B.R., Eberhart M.G., Brady K.A. (2013). Accuracy of definitions for linkage to care in persons living with HIV. J. Acquir. Immune Defic. Syndr..

[6] Samji H., Cescon A., Hogg R.S., Modur S.P., Althoff K.N., Buchacz K., Burchell A.N., Cohen M., Gebo K.A., Gill M.J. (2013). Closing the gap: Increases in life expectancy among treated HIV-positive individuals in the United States and Canada. PLoS ONE.

[7] Ministry of Health of the Russian Federation (2017). Clinical Guidelines “HIV Infection in Adults”. http://rushiv.ru/wp-content/uploads/2019/03/kr79.pdf.

[8] Ministry of Health of the Russian Federation (2020). Clinical Guidelines “HIV Infection in Adults”. http://rushiv.ru/wp-content/uploads/2022/11/KR79.pdf.

[9] Al-Salama Z.T. (2017). Elsulfavirine: First global approval. Drugs.

[10] International Treatment Preparedness Coalition Eastern Europe and Central Asia (2022). Analysis of Procurement of ARV Drugs in the Russian Federation in 2021.

[11] Federal Russian AIDS Center Report 2006. http://www.hivrussia.info/wp-content/uploads/2019/01/Byulleten-30-VICH-infektsiya-2006-g.pdf.

[12] Heath K., Levi J., Hill A. (2021). The Joint United Nations Programme on HIV/AIDS 95-95-95 targets: Worldwide clinical and cost benefits of generic manufacture. AIDS.

[13] Clutter D.S., Jordan M.R., Bertagnolio S., Shafer R.W. (2016). HIV-1 drug resistance and resistance testing. Infect. Genet. Evol..

[14] Standard of Primary Health Care for Adults with HIV Infection. http://rushiv.ru/wp-content/uploads/2022/07/0001202207260029.pdf.

[15] International Treatment Preparedness Coalition Eastern Europe and Central Asia (2022). Analysis of Procurement of Diagnostics for HIV Treatment in Russia in 2020–2021.

[16] Pokrovskaya A., Kireev D., Emerole K., Kirichenko A., Pokrovsky V. Pilot model of the HIV drug resistance testing coverage cascade. Proceedings of the HIV Glasgow Conference.

[17] Bennett D.E., Camacho R.J., Otelea D., Kuritzkes D.R., Fleury H., Kiuchi M., Heneine W., Kantor R., Jordan M.R., Schapiro J.M. (2009). Drug resistance mutations for surveillance of transmitted HIV-1 drug-resistance: 2009 update. PLoS ONE.

[18] Nosik M.N., Ryzhov K.A., Kravchenko A.V., Sevostyanihin S.E., Kuimova U.A., Potapova A.B., Sobkin A.L. (2019). Analysis of prevalence of HIV-1 primary resistance to antiretroviral drugs in the territory of Moscow and Moscow region. J. Microbiol. Epid. Immun..

[19] Antonova A.A., Tumanov A.S., Lebedev A.V., Kozyennova E.V., Glinkina L.N., Kulagin V.V., Shemshura A.B., Lebedev P.V., Khoteleva L.V., Bobkova M.R. (2022). Genetic profile and characteristics of HIV-1 drug resistance mutation in the Krasnodar region over the 2014 to 2019. HIV Inf. Immunosup. Disord..

[20] Kirichenko A.A., Kireev D.E., Lopatukhin A.E., Murzakova A.V., Lapovok I.A., Ladnaya N.N., Pokrovsky V.V. (2019). The level and structure of HIV-1 drug resistance among patients with no experience of taking antiretroviral drugs since the start of antiretroviral therapy in the Russian Federation. HIV Inf. Immunosup. Disord..

[21] Kirichenko A., Kireev D., Lopatukhin A., Murzakova A., Lapovok I., Saleeva D., Ladnaya N., Gadirova A., Ibrahimova S., Safarova A. (2022). Prevalence of HIV-1 drug resistance in Eastern European and Central Asian countries. PLoS ONE.

[22] Kirichenko A., Lapovok I., Baryshev P., van de Vijver D.A.M.C., van Kampen J.J.A., Boucher C.A.B., Paraskevis D., Kireev D. (2020). Genetic Features of HIV-1 Integrase Sub-Subtype A6 Predominant in Russia and Predicted Susceptibility to INSTIs. Viruses.

[23] Decree of the Chief State Sanitary Doctor of the Russian Federation of January 28, 2021 N 4 “On Approval of Sanitary Rules and Norms SanPiN 3.3686-21 “Sanitary and Epidemiological Requirements for the Prevention of Infectious Diseases””. https://www.rospotrebnadzor.ru/files/news/SP_infections_compressed.pdf.

[24] Stanford HIV Data Base. http://hivdb.stanford.edu.

[25] The Joint United Nations Programme on HIV/AIDS (2014). Understanding Fast-Track: Accelerating Action to End the AIDS Epidemic by 2030.

[26] World Health Organization (2017). HIV Drug Resistance Report.

[27] World Health Organization (2016). HIV Drug Resistance Surveillance Guidance—2015 Update.

[28] Wittkop L., Günthard H.F., de Wolf F., Dunn D., Cozzi-Lepri A., de Luca A., Kücherer C., Obel N., von Wyl V., Masquelier B. (2011). Effect of transmitted drug resistance on virological and immunological response to initial combination antiretroviral therapy for HIV (EuroCoord-CHAIN joint project): A European multicohort study. Lancet Infect. Dis..

[29] Hamers R.L., Schuurman R., Sigaloff K.C., Wallis C.L., Kityo C., Siwale M., Mandaliya K., Ive P., Botes M.E., Wellington M. (2012). Effect of pretreatment HIV-1 drug resistance on immunological, virological, and drug-resistance outcomes of first-line antiretroviral treatment in sub-Saharan Africa: A multicentre cohort study. Lancet Infect Dis..

[30] Phillips A.N., Stover J., Cambiano V., Nakagawa F., Jordan M.R., Pillay D., Doherty M., Revill P., Bertagnolio S. (2017). Impact of HIV drug resistance on HIV/AIDS-associated mortality, new infections, and antiretroviral therapy program costs in sub-Saharan Africa. J. Infect. Dis..

[31] Hofstra L.M., Sauvageot N., Albert J., Alexiev I., Garcia F., Struck D., Van de Vijver D.A.M.C., Åsjö B., Beshkov D., Coughlan S. (2016). Transmission of HIV Drug Resistance and the Predicted Effect on Current First-line Regimens in Europe. Clin. Infect. Dis..

[32] Cambiano V., Bertagnolio S., Jordan M.R., Lundgren J.D., Phillips A. (2013). Transmission of drug resistant HIV and its potential impact on mortality and treatment outcomes in resource-limited settings. J. Infect. Dis..

[33] Bobkov A., Kazennova E., Selimova L., Bobkova M., Khanina T., Ladnaya N., Kravchenko A., Pokrovsky V., Cheingsong-Popov R., Weber J. (1998). A sudden epidemic of HIV type 1 among injecting drug users in the former Soviet Union: Identification of subtype A, subtype B, and novel gagA/envB recombinants. AIDS Res. Hum. Retrovir..

[34] Foley B.T., Leitner T., Paraskevis D., Peeters M. (2016). Primate immunodeficiency virus classification and nomenclature: Review. Infect. Genet. Evol..

[35] Novitsky V.A., Montano M.A., Essex M. (1998). Molecular epidemiology of an HIV-1 subtype A subcluster among injection drug users in the Southern Ukraine. AIDS Res. Hum. Retrovir..

[36] Van de Klundert M.A.A., Antonova A., Di Teodoro G., Ceña Diez R., Chkhartishvili N., Heger E., Kuznetsova A., Lebedev A., Narayanan A., Ozhmegova E. (2022). Molecular Epidemiology of HIV-1 in Eastern Europe and Russia. Viruses.

[37] Kostaki E.G., Karamitros T., Bobkova M., Oikonomopoulou M., Magiorkinis G., Garcia F., Hatzakis A., Paraskevis D. (2018). Spatiotemporal Characteristics of the HIV-1 CRF02_AG/CRF63_02A1 Epidemic in Russia and Central Asia. AIDS Res. Hum. Retrovir..

[38] Baryshev P.B., Bogachev V.V., Gashnikova N.M. (2014). HIV-1 genetic diversity in Russia: CRF63_02A1, a new HIV type 1 genetic variant spreading in Siberia. AIDS Res. Hum. Retrovir..

[39] European Centre for Disease Prevention and Control (2019). Developing a Reporting System for the Surveillance of HIV Drug Resistance in Europe.

[40] Pasechnik O.A., Blokh A.I. (2018). The prevalence of HIV recombinant forms in Russia and countries of the CIS: Systematic review and meta-analysis. Rus. J. Infect. Immun..

[41] Moskaleychik F.F., Laga V.Y., Delgado E., Vega Y., Fernandez-Garcia A., Perez-Alvarez L., Kornilaeva G.V., Pronin A.Y., Zhernov Y.V., Thomson M.M. (2015). Rapid spread of the HIV-1 circular recombinant CRF02-AG in Russia and neighboring countries. Vopr. Virusol..

[42] Little S.J., Frost S.D., Wong J.K., Smith D.M., Pond S.L., Ignacio C.C., Parkin N.T., Petropoulos C.J., Richman D.D. (2008). Persistence of transmitted drug resistance among subjects with primary human immunodeficiency virus infection. J. Virol..

[43] Xu H.T., Colby-Germinario S.P., Asahchop E.L., Oliveira M., McCallum M., Schader S.M., Han Y., Quan Y., Sarafianos S.G., Wainberg M.A. (2013). Effect of mutations at position E138 in HIV-1 reverse transcriptase and their interactions with the M184I mutation on defining patterns of resistance to nonnucleoside reverse transcriptase inhibitors rilpivirine and etravirine. Antimicrob. Agents Chemother..

[44] Picchio G.R., Rimsky L.T., Van Eygen V., Haddad M., Napolitano L.A., Vingerhoets J. (2014). Prevalence in the USA of rilpivirine resistance-associated mutations in clinical samples and effects on phenotypic susceptibility to rilpivirine and etravirine. Antivir. Ther..

[45] Porter D.P., Toma J., Tan Y., Solberg O., Cai S., Kulkarni R., Andreatta K., Lie Y., Chuck S.K., Palella F. (2016). Clinical Outcomes of Virologically-Suppressed Patients with Pre-existing HIV-1 Drug Resistance Mutations Switching to Rilpivirine/Emtricitabine/Tenofovir Disoproxil Fumarate in the SPIRIT Study. HIV Clin. Trials.

[46] Kuznetsova A., Lebedev A., Gromov K., Kazennova E., Zazzi M., Incardona F., Sönnerborg A., Bobkova M. (2022). Pre-existing singleton E138A mutations in the reverse transcriptase gene do not affect the efficacy of first-line antiretroviral therapy regimens using rilpivirine in human immunodeficiency virus-infected patients. Clin. Case Rep..

[47] World Health Organization (2017). Global Action Plan on HIV Drug Resistance 2017–2021.

[48] World Health Organization (2017). Guidelines on the Public Health Response to Pretreatment HIV Drug Resistance.

[49] Miranda M.N.S., Pingarilho M., Pimentel V., Martins M.D.R.O., Kaiser R., Seguin-Devaux C., Paredes R., Zazzi M., Incardona F., Abecasis A.B. (2022). Trends of Transmitted and Acquired Drug Resistance in Europe From 1981 to 2019: A Comparison Between the Populations of Late Presenters and Non-late Presenters. Front. Microbiol..

