# Supplementary material for: HIV-1 Drug Resistance among Treatment-Naïve Patients in Russia: Analysis of the National Database, 2006–2022
Source: Viruses. 2023 Apr 18;15(4):991. doi: 10.3390/v15040991 (PMC10141655; doi:10.3390/v15040991)
Supplement: Supplementary file 1 [file viruses-15-00991-s001.zip › Table S1.pdf]

Table S1. Prevalence of DRMs in Russia in the 2006–2022 sampling years

Class of ART drugs	DRMs	Prevalence of DRMs, n (%)
PI	**L23I**	1 (0.02)
	**L24I**	1 (0.02)
	**D30N**	1 (0.02)
	V32I	1 (0.02)
	**M46I/L**	28 (0.6)
	**I47V**	3 (0.1)
	**I50L**	3 (0.1)
	**F53L/Y**	4 (0.1)
	**I54L**	1 (0.02)
	**G73S**	4 (0.1)
	**V82A**	2 (0.04)
	**I84V**	1 (0.02)
	**I85V**	14 (0.3)
	**N88D**	1 (0.02)
NRTI	**M41L**	9 (0.2)
	E44D	8 (0.2)
	A62V	1787 (39.9)
	**K65R**	2 (0.04)
	**D67N**	3 (0.1)
	**T69D**	3 (0.1)
	**K70R**	2 (0.04)
	**L74V**	2 (0.04)
	**V75M**	1 (0.02)
	**Y115F**	2 (0.04)
	**F116Y**	1 (0.02)
	**Q151M**	1 (0.02)
	**M184V/I**	30 (0.7)
	**L210W**	2 (0.04)
	**T215D/Y/S/I/E**	15 (0.3)
	**K219E**	1 (0.02)
NNRTI	A98G	9 (0.2)
	L100F	2 (0.04)
	**K101E**	17 (0.4)
	**K103N/S**	100 (2.2)
	V106I	57 (1.3)
	**V106M**	1 (0.02)
	E138A	239 (5.3)
	E138G/K/Q	30 (0.7)
	V179D/E/T	70 (1.6)
	**V179F**	1 (0.02)
	**Y181C**	10 (0.2)
	**Y188L/H**	4 (0.1)
	**G190A/S**	27 (0.6)
	H221Y	3 (0.1)
	**P225H**	1 (0.02)

	**M230L**	1 (0.02)
	K238T	3 (0.1)
INSTI	E92D	1 (0.1)
	Q146P	2 (0.2)
	Q148P	1 (0.1)
	V151L	1 (0.1)
	**R263K**	1 (0.1)

Surveillance drug resistant mutations are in bold. Abbreviations: DRMs, drug resistant mutations; PI, protease inhibitor; NRTI, nucleoside reverse-transcriptase inhibitor; NNRTI, non-nucleoside reverse-transcriptase inhibitor; INSTI, integrase strand transfer inhibitor.
