# Supplementary material for: HIV-1 Drug Resistance among Treatment-Naïve Patients in Russia: Analysis of the National Database, 2006–2022
Source: Viruses. 2023 Apr 18;15(4):991. doi: 10.3390/v15040991 (PMC10141655; doi:10.3390/v15040991)
Supplement: Supplementary file 1 [file viruses-15-00991-s001.zip › Table S2.pdf]

Table S2. HIV DR prevalence in FDs of Russia in the 2006–2022 sampling years by antiretroviral drugs

**Class of ART drugs**	**ART drug**	**FD**							
		Central (n=1661), %	Northwestern (n=297), %	Southern (n=666), %	North Caucasian (n=18), %	Volga (n=617), %	Ural (n=432), %	Siberian (n=353), %	Far Eastern (n=217), %
**Any**	Any	12.2	11.1	11.7	38.9	12.3	14.8	13.6	13.8
**NRTI**	ABC	1.0	0	0.3	0	0.8	2.1	1.4	1.8
	AZT	0.6	0.7	0.5	0	0.2	1.2	0.8	0.9
	d4T	0.7	0.7	0.5	0	0.5	0.9	1.4	0.9
	ddI	0.9	0	0.5	0	0.8	1.2	0.8	1.8
	FTC	0.7	0	0.3	0	0.5	1.6	1.1	1.8
	3TC	0.7	0	0.3	0	0.5	1.6	1.1	1.8
	TDF	0.4	0	0	0	0.3	0.5	0.6	0.5
**NNRTI**	DOR	1.6	1.7	0.9	0	0.8	3.5	0.3	0.9
	EFV	4.2	3.7	2.9	33.3	1.6	4.9	4.0	4.1
	ETR	1.3	1.0	0.5	0	0.5	2.1	1.1	0.9
	NVP	4.6	4.0	4.1	33.3	2.3	5.6	5.1	4.1
	RPV	7.2	6.4	6.9	0	7.9	9.5	5.4	9.2
**PI**	ATV	0.3	0.3	0	0	0.2	0.7	0	0
	DRV	0.1	0	0	0	0.2	0	0	0
	FPV	0.6	0.7	0.5	5.6	0.6	0.5	0	0.5
	IDV	0.5	0.7	0	0	0.3	0.2	0.3	0.9
	LPV	0.3	0.3	0	0	0.3	0.2	0	0
	NFV	1.8	2.0	0.8	5.6	2.4	2.5	1.7	1.4
	SQV	0.4	0.3	0	0	0.2	0.2	0.3	0.5
	TPV	0.5	0	0.3	0	0.3	0.5	0	0

Abbreviations: FD, Federal District.
