# Supplementary figures and images for: HIV-1 Drug Resistance among Treatment-Naïve Patients in Russia: Analysis of the National Database, 2006–2022
Source: Viruses. 2023 Apr 18;15(4):991. doi: 10.3390/v15040991 (PMC10141655; doi:10.3390/v15040991)

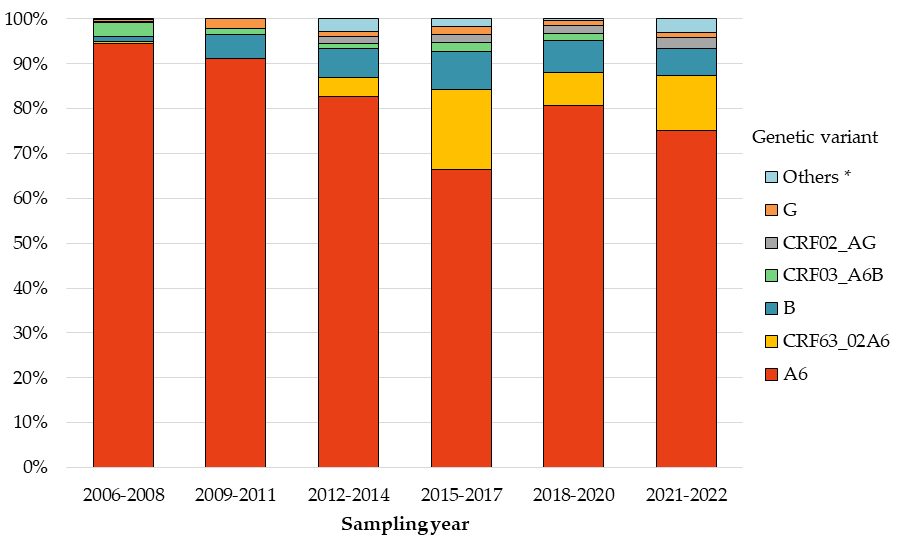

Supplement: Supplementary file 1 [file viruses-15-00991-s001.zip › Figure S1.tif]
